# Modulatory Mechanism of Nociceptive Neuronal Activity by Dietary Constituent Resveratrol

**DOI:** 10.3390/ijms17101702

**Published:** 2016-10-11

**Authors:** Mamoru Takeda, Shiori Takehana, Kenta Sekiguchi, Yoshiko Kubota, Yoshihito Shimazu

**Affiliations:** 1 Laboratory of Food and Physiological Sciences, Department of Life and Food Sciences, School of Life and Environmental Sciences, Azabu University, 1-17-71, Fuchinobe, Chuo-ku, Sagamihara, Kanagawa 252-5201, Japan; me1605@azabu-u.ac.jp (S.T.); che.4.mar29@gmail.com (K.S.); shimazu@azabu-u.ac.jp (Y.S.); 2 FANCL Health Science Research Center, Research Institute, FANCL corporation, 12-13, Kamishinano, Totsuka-ku, Yokohama, Kanagawa 244-0806, Japan; yoshiko_0909@fancl.co.jp

**Keywords:** resveratrol, trigeminal system, nociceptive neuron, hyperalgesia, complementary alternative medicine, trigeminal spinal nucleus, extracellular single unit recording

## Abstract

Changes to somatic sensory pathways caused by peripheral tissue, inflammation or injury can result in behavioral hypersensitivity and pathological pain, such as hyperalgesia. Resveratrol, a plant polyphenol found in red wine and various food products, is known to have several beneficial biological actions. Recent reports indicate that resveratrol can modulate neuronal excitability, including nociceptive sensory transmission. As such, it is possible that this dietary constituent could be a complementary alternative medicine (CAM) candidate, specifically a therapeutic agent. The focus of this review is on the mechanisms underlying the modulatory effects of resveratrol on nociceptive neuronal activity associated with pain relief. In addition, we discuss the contribution of resveratrol to the relief of nociceptive and/or pathological pain and its potential role as a functional food and a CAM.

## 1. Introduction

Resveratrol (*trans*-3,4′,5-trihydroxystilbene) is a dietary constituent and a plant polyphenol found in red wine and various food products [1,2]. It has been reported to have various beneficial actions, including anti-oxidative, anti-inflammatory, neuroprotective, anticancer, and cardioprotective effects [2,3,4,5,6]. Complementary and alternative medicines (CAM), such as herbal medicines and acupuncture, have been used to treat persistent clinical chronic pain [7,8,9], and considerable research has focused on the potential effects of diet and dietary supplementation on conditions associated with pain [10,11,12]. Resveratrol could be a CAM candidate for the treatment of pain.

Recent studies reported that resveratrol modulates neuronal excitability of the peripheral and central nervous systems (PNS and CNS, respectively) via various voltage-dependent [13,14,15,16] and ligand-gated [17,18] ion channels, including the sensory information processing system. Because resveratrol decreases the production of prostaglandin E_2_ (PGE_2_) by inhibiting the cyclooxygenase (COX)-2 cascade, resveratrol is a potent inhibitor of inducible COX-2 [19,20]. In fact, PGE_2_ is a well-known proinflammatory mediator and sensitizer of peripheral nociceptors that also acts on the CNS, including somatosensory neurons in the spinal dorsal horn [21,22,23]. Previous reports indicated that resveratrol inhibits inflammation-induced hyperalgesia by suppressing COX-1 and COX-2 activity [20,24,25]. It is well known that the analgesic action of the acidic antipyretic non-steroidal anti-inflammatory drugs (NSAIDs) involves the potent and efficient inhibition of COX-2 [26]. Together, these observations suggest that resveratrol could be used as a potential therapeutic agent for the prevention of inflammatory hyperalgesia.

The focus of this review is on the mechanism by which resveratrol modulates nociceptive neuronal activity, and its association with the relief of nociceptive and inflammatory pain. In addition, we introduce recent data and discuss the potential contribution of resveratrol to the relief of nociceptive and pathological pain, as well as its development as a functional food and CAM.

## 2. Trigeminal Pain Pathway and Spinal Trigeminal Nucleus

The trigeminal nervous system is known to have unique structures and functions for the processing of orofacial nociception as well as non-noxious sensations. The oral mucosal membrane, tongue, tooth pulp, gum, and temporomandibular joint (TMJ) are innervated by small-diameter Aδ-fibers and unmyelinated C-fibers that process orofacial nociception [27]. Noxious sensory information in the area innervated by the trigeminal nerves is relayed from trigeminal afferents to second-order neurons in the spinal trigeminal nucleus in the brainstem and the upper cervical (C1–C2) spinal cord [27,28]. The spinal trigeminal nucleus, an important relay station in the transmission of orofacial sensory information, is functionally subdivided into three nuclei: oralis, interpolaris, and caudalis [27]. In addition to the C1–C2 dorsal horn, the spinal trigeminal nucleus caudalis (SpVc) is a relay station for trigeminal nociceptive inputs from inflammation and tissue injury [27,28]. The properties of somatic sensory pathways can be altered by chronic pathological conditions, such as tissue inflammation, leading to hyperalgesia and allodynia [29]. Information processing in the spinal trigeminal nucleus or higher centers is altered by changes in the excitability of primary afferent neurons, which is known as peripheral sensitization [30]. Because it has been reported previously that wide dynamic range (WDR) neurons in the SpVc play an important role in hyperalgesia, allodynia, and/or referred pain associated with orofacial pain [31,32,33,34], in the following sections we focus on studies of the SpVc WDR neuronal activity in the trigeminal pain pathway.

## 3. Potential Role for Resveratrol in Alleviating Nociceptive Pain

### 3.1. Peripheral Mechanism

There are four general processes involved in nociceptive sensory signaling: (1) transduction of external stimuli from peripheral terminals; (2) action potential generation; (3) action potential propagation along axons; and (4) transmission to central terminals that form the presynaptic elements of the first synapses in sensory pathways in the CNS [28,35]. Resveratrol has been reported to modulate the excitability of neurons in the PNS by activating voltage-dependent and transient receptor potential (TRP) channels [13,14,15,16,36]. In vitro, mechanical stimuli have been shown to induce mechanosensitive currents via mechanosensitive channels, such as TRP ankyrin 1 (TRPA1), triggering mechanotransduction in trigeminal neurons innervating the inner walls of the anterior eye chamber [37]. In addition, TRPA1 modulates mechanotransduction in primary sensory neurons [38]. Potent inhibition of TRPA1 in vitro and in vivo by resveratrol [36] suggests that resveratrol attenuates the generator potential and inhibits action potential firing via the mechanical transduction process. Moreover, resveratrol has been shown to modulate Na^+^ and K^+^ currents in dorsal root ganglion (DRG) neurons associated with action potential generation [13,16], and that resveratrol predominantly inhibits Na^+^ currents in acutely dissociated DRG neurons, indicating inhibition of action potential generation [13]. 

**Figure 1:**
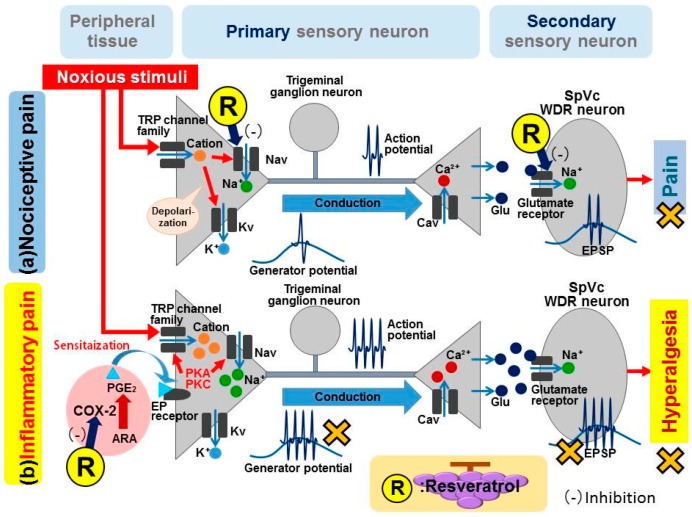
Schematic drawing of the possible mechanism underlying the effects of resveratrol in relieving (**a**) nociceptive and(**b**) inflammatory pain. (**a**) nociceptive pain. When noxious mechanical stimulation is applied to the skin, mechanosensitive ion channels (e.g., transient receptor potential ankyrin 1 (TRPA1) channels) open, activating the generator potential (depolarization). This depolarization further opens voltage-dependent sodium and potassium channels, generating action potentials. Action potentials are discharged through primary afferent fibers (Aδ- and C-fibers) to the central terminal in nociceptive neurons in the trigeminal spinal nucleus caudalis (SpVc). When action potentials are conducted to the central terminal of the SpVc, presynaptic voltage-dependent calcium channels open, leading to the release of neurotransmitters (e.g., glutamate) into the synaptic cleft, which then bind to post-synaptic (glutamate) receptors, activating excitatory post-synaptic potentials (EPSP). If the amplitude of EPSPs is over the action potential threshold, a barrage of action potentials is conducted to higher centers in the pain pathway and pain is perceived. It is possible that resveratrol suppresses both the excitability of peripheral terminals of the trigeminal nerve (by modulating both the mechanical transduction and generation of action potentials) and glutaminergic excitatory synaptic transmission of the SpVc (by inhibiting post-synaptic glutamate receptors and presynaptic Ca^2+^ channels). R, resveratrol; Nav, voltage-gated sodium channel; Kv, voltage-gated potassium channel; Cav, voltage-gated calcium channel; Glu, glutamate; WDR, wide dynamic range neurons; (**b**) Inflammatory pain. Following peripheral inflammation and/or nerve injury, inflammatory mediators, such as prostaglandin E_2_ (PGE_2_), bind to G-protein-coupled E-type prostanoid (EP) receptors and induce activation of protein kinases A and C (PKA and PKC, respectively) in nociceptive peripheral terminals, leading to phosphorylation of mechanosensitive, sodium and potassium ion channels and receptors. As a result, the activation threshold for transducer channels such as TRPA1 is reduced and the membrane excitability of the peripheral terminals increases, resulting in a high frequency of action potentials being conducted to presynaptic central terminals of the SpVc. This results in the release of a large amount of glutamate into the synaptic cleft, which binds to upregulated post-synaptic glutamate receptors, augmenting EPSPs, causing a barrage of action potentials to be conducted to higher centers of pain pathways and creating a state of heightened sensitivity termed peripheral sensitization. It is possible that chronic administration of resveratrol attenuates inflammation-induced mechanical inflammatory hyperalgesia, with this effect due primarily to suppression of the hyperexcitability of SpVc WDR neurons via inhibition of both peripheral and central cyclooxygenase (COX)-2 cascade signaling pathways. ARA, arachidonic acid. X: Suppression.

Recently, the effects of local subcutaneous injection of resveratrol into the receptive field of SpVc WDR neurons on non-noxious and noxious mechanical stimulation-induced excitability of these neurons were investigated in vivo [39]. In that study, the mean firing rate of SpVc WDR neurons in response to both non-noxious and noxious mechanical stimuli was dose-dependently reduced by resveratrol; this effect was reversible and the mean magnitude of inhibition of the SpVc neuron discharge frequency was almost equal between resveratrol and the local anesthetic lidocaine (1%). These observations suggest that resveratrol injection into the peripheral receptive field suppresses SpVc neuron excitability, possibly by inhibiting Na^+^ channels in the nociceptive nerve terminals of trigeminal ganglion neurons. Thus, it may be that resveratrol inhibits the excitability of peripheral terminals of the trigeminal nerve by modulating both the noxious mechanical stimulation-induced generator potential and the initiation of action potential processes (Figure 1).

### 3.2. Central Mechanism

In hippocampal slices, resveratrol has been shown to significantly suppress glutamate-induced currents in post-synaptic CA1 pyramidal neurons without having any presynaptic effects [17]. In addition, Gao et al. [17] indicated that *N*-methyl-d-aspartate (NMDA) receptors were more sensitive to resveratrol than α-amino-3-hydroxy-5-methyl-4-isoxazole proprionic acid (AMPA) receptors. It has also been reported that action potential duration and l-type Ca^2+^ currents in ventricular myocytes are reduced by resveratrol [14]. Thus, resveratrol may suppress glutamatergic excitatory synaptic transmission of the SpVc by inhibiting post-synaptic glutamate receptors and presynaptic Ca^2+^ channels (Figure 1).

Recently, the effects of acute intravenous administration of resveratrol to rats on the excitability of nociceptive WDR SpVc neuronal activity in vivo in response to mechanical stimulation were investigated [40]. In that study, the mean SpVc WDR neuronal firing rate in response to both non-noxious and noxious mechanical stimuli was dose-dependently inhibited by resveratrol in a reversible manner, and the relative magnitude of inhibition by resveratrol of the SpVc WDR neuronal discharge frequency was significantly greater for noxious than non-noxious stimuli. These findings suggest that, in vivo, trigeminal nociceptive transmission in the SpVc is suppressed by acute intravenous resveratrol at the level of secondary neurons, in addition to primary neurons.

The dose-dependent antinociceptive effects of systemic resveratrol appear to be mediated via an opioidergic mechanism, because naloxone pretreatment of rats completely blocked the analgesic effect of resveratrol [41]. Evoked inhibitory GABAergic pre- and post-synaptic potentials in the periaqueductal gray (PAG) are partially inhibited by opiates acting via μ-opioid receptors [42]. Neuronal activity in the PAG is increased after blockade of μ-opioid receptors as a result of GABAergic disinhibition, resulting in the subsequent activation of serotonergic (5-hydroxytrypytamine (5-HT)) neurons in the nucleus raphe magnus, known as the PAG–nucleus raphe magnus–trigeminal pathway [43,44]. Resveratrol has been shown to facilitate 5-HT_3_ receptor-mediated ion currents [18], and nociceptive stimulation-evoked SpVc/C1 neuron activity is suppressed by conditioning peripheral nerve stimulation via 5-HT_3_ receptor-mediated GABAergic inhibition [45,46]. Together, these observations suggest that resveratrol suppresses excitatory synaptic transmission of the SpVc via 5-HT_3_ receptor-mediated GABAergic inhibition and/or endogenous opioidergic mechanisms. However, further studies are needed to elucidate the precise mechanisms involved.

## 4. Potential Role for Resveratrol in Alleviating Inflammatory Pain

It has been reported that peripheral tissue injury or inflammation of the innervating trigeminal nerve can alter the properties of trigeminal somatic sensory pathways, causing behavioral hypersensitivity and resulting in increased responses to pain caused by noxious stimuli (e.g., hyperalgesia) [29]. 

Because it has been shown that resveratrol inhibits COX-1 and COX-2 activity [20,24,25], we recently tested the hypothesis that chronic administration of resveratrol would attenuate inflammation-induced hyperexcitability of trigeminal nociceptive neuronal activity associated with hyperalgesia in behavioral and electrophysiological experiments [47]. In that study, the threshold of escape from mechanical stimulation applied to the orofacial area in inflamed rats was significantly lower than in naïve rats, and the lowered mechanical threshold in inflamed rats was returned to control levels following chronic administration of resveratrol. In addition, after resveratrol administration, the mean discharge frequency of SpVc WDR neurons in inflamed rats was significantly decreased in response to both non-noxious and noxious mechanical stimuli, and significant decreases were observed in the inflammation-induced increased spontaneous discharge of SpVc WDR neurons and the frequency and occurrence of noxious pinch-evoked after discharge [47]. Finally, resveratrol administration restored the expanded receptive field of inflamed rats to control levels [47]. These results suggest that chronic administration of resveratrol attenuates inflammation-induced mechanical inflammatory hyperalgesia and that this effect is due primarily to the suppression of the hyperexcitability of SpVc WDR neurons via inhibition of both peripheral and central COX-2 cascade signaling pathways. These findings support the idea that resveratrol could be used as a potential therapeutic agent, or CAM, for the prevention of trigeminal inflammatory hyperalgesia.

It is known that PGE_2_ appears to facilitate the activation of TRP vanilloid 1 (TRPV1) and tetrodotoxin (TTX)-resistant (TTX-R) Na^+^ channels [13,29,48], and it has been reported that resveratrol inhibits both TTX-sensitive (TTX-S) and TTX-R Na^+^ currents in acutely dissociated DRG neurons. It appears that TTX-R Na^+^ channels (e.g., Nav1.8 and Nav1.9) are selectively expressed in small- and medium-sized DRG neurons [49]. These small DRG neurons are somata that give rise to thinly and unmyelinated C- and Aδ-fibers, which primarily conduct nociceptive stimuli. These Na^+^ channels can be modulated by activation of adenylate cyclase and increases in cAMP, possibly leading to protein kinase A-dependent phosphorylation of the Na^+^ channels. In this way, PGE_2_ produced during an inflammatory response may significantly increase the excitability of nociceptive fibers (peripheral sensitization). Because it has been reported that the increased excitability of small-diameter trigeminal ganglion neurons seen after PGE_2_ application involves increases in TTX-R Na^+^ currents [50], it can be assumed that resveratrol inhibits the excitability of small-diameter trigeminal ganglion neurons by suppressing TTX-R Na^+^ currents induced by the increased production of PGE_2_. It seems reasonable to speculate that at least part of the peripheral antinociceptive action of resveratrol arises as a result of the prevention of peripheral sensitization, as is the case for antipyretic analgesics (Figure 1).

Conversely, PGE_2_ can also act in the CNS, namely in the spinal dorsal horn and SpVc neurons, to produce hyperalgesia [21]. Inflammation-induced increases in COX-2 mRNA and protein have been demonstrated in the spinal cord [23,51], where COX-1 and COX-2 are expressed constitutively. Recent evidence indicates that a major stimulus for the induction of COX-2 is the proinflammatory cytokine interleukin-1β, which is found in the periphery as well as in the CNS and is produced in response to inflammation [23,52]. Two possible molecular mechanisms have been proposed to account for PGE_2_-induced hyperalgesia via actions in the CNS: (1) PGE_2_ reduces inhibitory glycinergic neurotransmission via a post-synaptic mechanism [53]; and (2) direct depolarization of deep dorsal horn neurons by higher concentrations of PGE_2_ [22]. Therefore, it is most likely that systemic administration of resveratrol has central antinociceptive effects by suppressing PGE_2_-induced reductions in inhibitory glycinergic neurotransmission and enhanced depolarization of SpVc neurons through a post-synaptic mechanism. However, further studies are needed to confirm this hypothesis.

## 5. Functional Significance of Pain Relief by Resveratrol and Future Directions

A widely accepted trigeminal chronic pain model is the complete Freund’s adjuvant (CFA) inflamed rat model [54,55]. Changes in neuronal properties resulting from tissue injury and inflammation of the area innervating the orofacial area can lead to pathological pain, including hyperalgesia and allodynia [33,54]. In a previous study, we reported that TMJ inflammation-induced hyperexcitability of SpVc WDR neurons innervating the facial skin, contributes to ectopic mechanical allodynia of this area [32,33]. Moreover, resveratrol restored the increased mean spontaneous discharge frequency of SpVc WDR neurons in inflamed rats to control levels [47]. Burnstein et al. [56] reported that an ongoing headache (spontaneous pain) is caused by ongoing activity in the SpVc. The origin of this ongoing activity in the central neurons that relay sensory information is of considerable clinical interest, because it has been suggested that it determines the level of post-traumatic injury and chronic pain [57]. More recently, it was shown that the ongoing activity of WDR neurons in the SpVc is driven from the periphery, because microinjection of lidocaine into trigeminal ganglia significantly decreases ongoing activity [58]. Because chronic administration of resveratrol attenuates the spontaneous activity in inflamed rats, these observations together suggest that resveratrol attenuates the spontaneous discharge activity (due to peripheral and/or trigeminal ganglion sensitization and probably contributing to spontaneous pain) of SpVc WDR neurons innervating the facial skin [59].

Although a previous study indicated that dietary grape seed polyphenol extract inhibited TMJ inflammation-induced pain [60], little is known regarding the mechanism by which polyphenols suppress nociceptive neuronal activity. More recently, we reported that in the absence of inflammatory or neuropathic pain, acute intravenous administration of resveratrol suppresses trigeminal sensory transmission, including nociception [40], and so resveratrol may contribute to the suite of CAMs as a therapeutic agent, for the treatment of trigeminal nociceptive pain [7]. In a previous study, under in vivo conditions, systemic administration of resveratrol attenuated inflammation-induced hyperexcitability of trigeminal SpVc neurons associated with hyperalgesia in rats [47]. Recently, there has been increased interest in the use of CAM for the treatment of persistent chronic pain [9,61]. Patients frequently turn to CAM for pain control when other medical treatments are ineffective [8,9]. Recent studies have also focused on the potential effects of diet and dietary supplementation on conditions associated with pain [10,11,62].

Because surgical incisions cause acute pain, and surgery is a potential cause of chronic pain [63,64], it is possible that resveratrol could effectively reduce clinical pain, including postoperative pain [65,66]. In patients with trigeminal neuralgia, local and intravenous administration of lidocaine has been reported to effectively attenuate pain intensity, including allodynia and hyperalgesia [67,68,69]. The results of the different studies into resveratrol contribute to the development of analgesic drugs with fewer side effects for the treatment of pathological pain, including orofacial pain. In particular, the findings from in vivo studies support the idea that resveratrol is a potential therapeutic agent that could be used as an alternative to alleviate nociceptive pain and prevent trigeminal inflammatory hyperalgesia.

## 6. Concluding Remarks

Recent studies provide evidence that: (1) local resveratrol injection into the peripheral receptive field suppresses the SpVc neuron excitability, possibly by inhibiting generator and action potentials in the nociceptive nerve terminals of trigeminal ganglion neurons; (2) trigeminal sensory transmission, including nociception, is suppressed by acute intravenous resveratrol; and (3) chronic administration of resveratrol attenuates inflammation-induced mechanical hyperalgesia, and this effect is due primarily to the suppression of hyperexcitability of SpVc WDR neurons via inhibition of peripheral and central COX cascade signaling pathways. Together, these findings support the idea that resveratrol may be a potential therapeutic CAM for the alleviation of nociceptive pain and prevention of trigeminal inflammatory hyperalgesia. The pain relief afforded by resveratrol appears to involve modulation of nociceptive neuronal activity.
